# Self-reported sexual coercion among in-school young people with disabilities in Ghana

**DOI:** 10.1186/s12889-024-18631-6

**Published:** 2024-05-03

**Authors:** Abdul-Aziz Seidu, Akwasi Kumi-Kyereme, Eugene K. M. Darteh

**Affiliations:** 1 Department of Population and Health, University of Cape Coast, Cape Coast, Ghana; 2 College of Public Health, Medical and Veterinary Sciences, James Cook University, Townsville, Australia

**Keywords:** Disability, Ghana, Prevalence, Sexual coercion, Young people

## Abstract

**Background:**

Sexual coercion is one of the major public health concerns globally. This is even more worrying among young people with disabilities (YPWDs). This study assessed the prevalence and factors associated with sexual coercion among in-school young people with disabilities in Ghana.

**Methods:**

Using a cross-sectional study design, pre-tested questionnaires were used to collect data from 979 YPWDs in 15 special schools for the visually and hearing impaired in Ghana. Sexual coercion was the outcome variable. Both descriptive (frequencies and percentages) and inferential analysis (binary logistic regression) were conducted.

**Results:**

About 68% reported that they had been sexually coerced at some point in their lifetime. This was higher among males (69.9%) compared to females (66.8%). Those aged 15–19 (72.19%) had the highest prevalence compared to those aged 20–24 (61.74%). YPWDs in Junior High School [JHS] [aOR = 1.722; CI = 1.227,2.417], and those in the coastal zone [aOR = 1.616; CI = 1.068,2.443] had higher odds of being coerced. However, those belonging to the Islamic religion [aOR = 0.266; CI = 0.0764,0.928] and the visually impaired [aOR = 0.477; CI = 0.318,0.716] had lower odds of being coerced compared to those with no religion, and the hearing impaired, respectively.

**Conclusion:**

There is a relatively high prevalence of sexual coercion among in- school YPWDs in Ghana. This is significantly associated with level of education, ecological zone, religion, and the type of disability. This calls for a concerted effort by policy makers such as the Ghana Education Service, Ghana Federation of the Disabled, Ministry of Education, Ministry of Gender, Children and Social Protection to intensify sex education and put in pragmatic steps to halt this serious public health issue.

## Background

Globally, approximately 16% of the world’s population comprises persons with disabilities [1]. From this group, about 80% face significant challenges in their daily activities [1]. The prevalence of disabilities is higher in low-and middle-income countries compared to high-income countries [1]. In Ghana, approximately 8% of the population lives with disabilities [2]. Persons with disabilities are recognized as one of the marginalized groups facing diverse challenges, with young people with disabilities (YPWDs) experiencing even more pronounced difficulties [3]. Worldwide, sexual coercion is a significant issue perpetrated against YPWDs [4–6]. It is defined as the act of forcing someone into a sexual act against their will [7]. Essentially, it involves the use of power to engage in sexual intercourse with a less powerful individual, usually without a formal agreement between the parties involved. In this study, it is regarded as sexual activity against a young person’s will. The World Health Organization (WHO) reports that the majority of sexually coerced survivors and most sexual violence cases involve people with disabilities with YPWDs being more susceptible [8].The prevalence of sexual coercion among young people varies globally, ranging from 14.8% in Botswana [9] to 25.2% in Ghana [10]. Among those with disabilities, the prevalence ranges from 24.6% in Nigeria [11] to 31% in Canada [6].

Coerced sex perpetrated against YPWDs could expose them to various adverse health outcomes [12]. For example, in cases where YPWDs cannot negotiate for safer sex, they are at a higher risk of being exposed to sexually transmitted infections including HIV/AIDS, gonorrhoea, syphilis, among others [13]. Additionally, female YPWDs could be exposed to gynaecological complications, including excruciating pain due to injured reproductive organs, bleeding, perforation of the uterus, infertility, painful menstruation, pelvic pain, and genital infections and discharges [14]. Apart from the physical consequences, survivors, their families, and neighbours may experience long-term psychological trauma [14, 15]. For instance, survivors of sexual coercion may experience depression and suicidal tendencies [16, 17]. The danger of sexual coercion and prejudice among YPWDs is influenced by factors such as poverty, gender inequality, and disability, indicating that in-school YPWDs, especially girls, may be particularly at risk [18]. However, existing research often overlook gender disparities or gather data on the identity of those who commit acts of violence. In fact, only 12 of the 17 studies included in a systematic review stated who the perpetrator was [18].

Previous studies on sexual coercion among students have been conducted in Canada [6], Ethiopia [19, 20], Nigeria [21], Botswana [9] and Ghana [10, 22, 23]. Most of these studies are limited in scope or considered the general tertiary student population. For example, those in Ghana focused on tertiary students [22, 23] or adolescents [10]. The few studies conducted among students with disabilities have focused only on those in tertiary institutions [24]. However, those who are more vulnerable are those in basic and secondary schools [10]. Also, among the general population, the available studies only looked at sexual violence against women with disabilities [25] and coerced first sexual intercourse among Ghanaian women [26].

Despite the documented detrimental effects of coerced sex towards YPWDs, there is limited research focus on this phenomenon in the Ghanaian context, especially among YPWDs who are already considered a marginalized group [27]. Moreover, having a disability sometimes puts a person at greater risk of sexual coercion than persons without disabilities due to dependence on others for some daily activities [5]. This study, therefore, aims to fill these gaps by examining the prevalence of sexual coercion among in-school YPWDs and its associated factors in Ghana. The findings are expected to inform policy and practice in the Ghanaian education sector and guide the development of pragmatic strategies to address sexual coercion against YPWDs.

### Theoretical framework

The study was situated in the Bronfenbrenner’s [28] ecological systems model. The model has been used in different disciplines since it was first conceptualised to understand the relationship between human growth and behaviour in their environment [29, 30]. The model argue that people are nested within a group of four interrelated systems, including microsystems, mesosystems, ecosystems, and macrosystems [31]. These hierarchical systems are connected in a way that the macro system ultimately determines how an individual acts or behaves toward an object or referent by interacting with the mesosystem, which also turn to interacts and informs the ecosystem [32]. These systems have a significant impact on how young people interact with one another and behave in specific situations [31, 32].

A person’s interactions with their social ecology or the milieu in which they find themselves directly influence how they involve or experience certain sexual behaviours [32]. Demographic characteristics and traits of an individual are included at the microsystem level. In this study the immediate social context, including the peer group, fellow students, the school, the family, and self-reported religious affiliation of the YPWD, could be referred to as the mesosystem. It could also refer to the social setting in which a young person participates in school activities with their peers as well as school-related administrative concerns such the regulations and laws governing sexual behaviour and the kinds of extracurricular activities. Lastly, the macrosystem refers to the wide societal elements that determine how young people will engage in sexual activity, such as region or ecological zones, socioeconomic position, cultural ideas, and practices connecting young people’s sexual behaviour, and the influence on how they will act sexually. YPWDs experience of sexual coercion is an example of a complicated, multi-dimensional phenomenon where a number of factors interact [32].

## Methods

### Ethical considerations

The study obtained ethical approval from the University of Cape Coast Institutional Review Board (UCCIRB/EXT/2017/13). In the Informed Consent Form, respondents were informed of the study’s goal, confidentiality, and the right to decline or withdraw at any time. Permission was also obtained from the heads of all the sampled schools. Both verbal and written informed consent was obtained from the respondents. For those who were minors, their legal guardians (the heads of the schools) also granted their approval before the minors assented. The procedure of obtaining heads of schools’ consent was approved by the ethical committee. The study adhered to all the ethical procedures as outlined in the Helsinki declaration.

### Study design and sampling

The data used for this present study forms part of a larger project in Ghana titled *“Sexual and Reproductive Health and Leisure Needs of Young People with Disabilities in Ghana” supported by the Directorate of Research, Innovation and Consultancy (DRIC), University of Cape Coast*. The larger project employed cross-sectional study design to collect quantitative data from YPWDs across the 16 administrative regions of Ghana (previously 10 regions). These administrative regions are strategically distributed across three overarching ecological zones, namely the Coastal, the Middle (forest belt), and the Northern (savannah) regions. The data collection period spanned from November 1 to December 22, 2017 [33, 34].

Out of the 35 special schools in Ghana, the study purposively sampled 16 schools catering to the visually impaired and the hearing impaired, with 14 schools for the hearing impaired and two schools for the visually impaired. One of the sampled schools declined to participate. The inclusion criteria in the study were: (a) being a student in the special school for the visually impaired or visually impaired, (b) students aged between 10 and 24 years (c) having either a visual impairment or hearing impairment, and (d) providing informed consent/assent to participate in the survey. The exclusion criteria were students with multiple disabilities, students less than 10 years or above 24 years of age and those who did not consent to participate in the survey. The total enrolment of students in the 15 schools was 4,180. From this number those that were eligible to participate in the study were 2,840 however 2,114 (74.4%) of them consented to participate in the study. The number of male pupils or students who participated in the study was more (1163;55.0%) than the females (951;45.0%). More than half (54.8%) of the respondents were in the middle ecological zone [33, 34]. However, for this present study 979 YPWDs who indicated that they have ever had sex constituted the study sample.

### Methods of data collection

After obtaining approval from the heads of schools during the data collection, information about the study captured in the information sheets was shared with eligible students in their classrooms. Questionnaires, including a braille version for the visually impaired, were administered separately to visually impaired and hearing impaired in different classrooms, allowing time for responses to each question before proceeding to the next. The adapted questionnaire was pre-tested in a special inclusive school in Cape Coast. Two sections of the questionnaire (socio-demographic characteristics of the respondents and sexual coercion) sections were used for this study.

The respondents were guided to self-respond to the questionnaire. They were given time to respond to each question after it had been explained before the next question. Three field assistants, selected for their expertise in special education and knowledge of sexual and reproductive health related issues, were engaged and trained for two days before the questionnaire pre-testing and the actual data collection. The team included a certified sign language interpreter and two Master of Philosophy students from the Departments of Population and Health and Special Education at the University of Cape Coast, Ghana. Details of the study methodology is published previously [33, 34].

### Study variables

The outcome variable for the study was sexual coercion. It was derived from the question “The first time you had sexual intercourse, would you say you had it because you wanted to, or because you were forced to have it against your will? [22] The responses were: a) I forced, b) I persuaded, c) Boy/girlfriend persuaded, d) Boy/girlfriend forced, and e) Both willing. These were transformed to derive a dichotomous variable, where a-d were categorised as sexually coerced (1) and not sexually coerced (0). The independent variables comprised: sex (Male, female), age (years) (10–14, 15–19, 20–24), level of education (Primary, Junior High School [JHS], Senior High School/Technical/Commercial), Religion (No religion, Christianity, Islam), ecological zone (Northern, Middle, Coastal), and disability type (hearing impaired, visually impaired).

### Data analyses

The study employed both descriptive and inferential statistics to analyse the data. Specifically, frequencies and percentages were used for the descriptive analysis. This was used to describe the socio-demographic characteristics of the respondents and the prevalence of sexual coercion. Chi-square analysis was also employed to determine whether there were statistically significant differences in the prevalence of sexual coercion across the independent variables. At the inferential level, binary logistic regression analysis was conducted to assess the factors associated with sexual coercion. The results were presented as crude odds ratios and adjusted odds ratios (aOR) and their corresponding 95% confidence intervals. Statistical significance was pegged at *p* < 0.05. All analysis were conducted using Stata version 14.

## Results

### Background characteristics and prevalence of sexual coercion among young people with disabilities

As shown in Table 1, the prevalence of sexual coercion was 68.6%. This was higher among males (69.9%) compared to females (66.8%). The chi-square analysis also showed that age(*p* = 0.011), educational level (*p* < 0.001), ecological zone(*p* = 0.014) and disability type (*p* < 0.001), are associated with experience of sexual coercion (Table 1).

**Table 1:** Background characteristics and prevalence of sexual coercion among young people with disabilities

Variable	Frequency	Percentage	Sexually coerced		X^2^(*p*-value)
			No n (%)	Yes n(%)	
**Sex**					1.00 (0.316)
Male	584	59.65	176 (30.14)	408 (69.86)	
Female	395	40.35	131 (33.16)	264 (66.84)	
**Age (Years)**					9.09 (0.011)
10–14	170	17.36	58 (34.12)	112 (65.88)	
15–19	579	59.14	161 (27.81)	418 (72.19)	
20–24	230	23.49	88 (38.26)	142 (61.74)	
**Education**					20.7 (< 0.001)
Primary	305	31.15	109 (35.74)	196 (64.26)	
JHS	499	50.97	125 (25.05)	374 (74.95)	
SHS/Tec/comm	175	17.88	73 (41.71)	102 (58.29)	
**Religion**					5.6 (0.062)
No religion	25	2.55	4 (16.00)	21 (84.00)	
Christianity	796	81.31	244 (30.65)	552 (69.35)	
Islam	158	16.14	59 (37.34)	99 (62.66)	
**Ecological zone**					8.5 (0.014)
Northern	248	25.33	90 (36.29)	158 (63.71)	
Middle	503	51.38	162 (32.21)	341 (67.79)	
Coastal	228	23.29	55 (24.12)	173 (75.88)	
**Disability type**					13.9 (< 0.001)
Hearing impaired	852	87.03	249 (29.23)	603 (70.77)	
Visually impaired	127	12.97	58 (45.67)	69 (54.33)	
**Total**	**979**	**100.0**	**307 (31.36)**	**672 (68.64)**	

*JHS* Junior High School, *SHS* Senior High School

### Factors associated with sexual coercion among young people with disabilities in Ghana

Table 2 shows the results from the regression analysis on the factors associated with sexual coercion among YPWDs in Ghana. Those in JHS [aOR = 1.722;95%CI = 1.227,2.417], and those in the coastal zone [aOR = 1.616; CI = 1.068,2.443] had higher odds of being coerced. On the other hand, those belonging to the Islamic religion [aOR = 0.266;95%CI = 0.0764,0.928] and the visually impaired [aOR = 0.477; 95%CI = 0.318,0.716] had lower odds of being coerced compared to those with no religion, and the hearing impaired respectively.

**Table 2:** Factors associated with sexual coercion among young people with disabilities in Ghana

	Model I	Model II
Variable	cOR [95%CI], *p*-value	aOR [95%CI], *p*-value
**Sex**		
Male	Ref	Ref
Female	0.869 [0.661,1.144], 0.317	0.869 [0.654,1.154], 0.332
**Age (Years)**		
10–14	Ref	Ref
15–19	1.344 [0.933,1.938],0.113	1.134 [0.767,1.677], 0.530
20–24	0.836 [0.552,1.264], 0.395	0.769 [0.481,1.228], 0.272
**Education**		
Primary	Ref	Ref
JHS	1.664 [1.221,2.268], 0.001	1.722 [1.227,2.417], 0.002
SHS/Tec/com/voc	0.777 [0.531,1.138], 0.195	0.799 [0.504,1.266], 0.339
**Religion**		
No religion	Ref	Ref
Christianity	0.431 [0.146,1.269], 0.127	0.329 [0.0971,1.116], 0.074
Islam	0.320 [0.105,0.977], 0.045	0.266 [0.0764,0.928], 0.038
**Ecological zone**		
Northern	Ref	Ref
Middle	1.199 [0.871,1.650], 0.266	1.356 [0.954,1.929], 0.090
Coastal	1.792 [1.202,2.670], 0.004	1.616 [1.068,2.443], 0.023
**Disability type**		
Hearing impaired	Ref	Ref
Visually impaired	0.491 [0.336,0.718], < 0.001	0.477 [0.318,0.716], < 0.001
**N**	**979**	**979**

Model II adjusted for sex, age, education, religion, ecological zone and disability type

*cOR* Crude odds ratio, *aOR* adjusted odds Ratio, *Ref* Reference Category, *JHS* Junior High School, *SHS* Senior High School, *Tec* Technical, *Com* Commercial, *Voc* Vocational

## Discussion

Sexual violence is a major public health concern globally. This study assessed the prevalence and factors associated with sexual coercion among in-school YPWDs. The results showed that about 68 out of every 100 in-school YPWDs who participated in this study have ever been sexually coerced. Although, males had relatively higher prevalence of experiencing sexual coercion than females, both the chi-square and the regression analysis did not show any statistically significant associations. This is similar to a previous study conducted by Gbagbo et al. [24] among tertiary students with disabilities. However, the findings are contrary to what was found by Keetile and Rakgoasi [9] among adolescents in Botswana that females were more likely to experience sexual coercion than males. They argued that in general females are usually the survivors of gender-based violence and sexual harassment [35]. In our study, the males were more than the females, this could also be a reason for the high prevalence.

Overall, the prevalence of sexual coercion is similar to previous studies that showed high sexual coercion among people with disabilities [4–6, 15, 36]. A possible reason for this finding may be because of the stereotypical perception and misconception that people with disabilities are less powerful or easily targeted for indiscriminate sexual behaviours [4–6, 15, 36]. For instance, individuals with disabilities may be less likely to defend themselves from sexual coercion and may be less likely to recognise and prevent any possible sexual coercive strategies [6, 37, 38]. This finding calls for policy makers to implement stringent measures such as prosecution to deter habitual perpetrators and people who intend to sexually coerced YPWDs.

YPWDs in JHS had higher odds of being sexually coerced compared to their counterparts in primary school. The probable explanation is that most of those in JHS might be more sexually matured compared to those in primary schools. A previous study by Jahanfar et al. [39] corroborates this finding. Jahanfar et al. [39] also argued that YPWDs in JHS are more knowledgeable about sexual and reproductive health issues including sexually transmitted infections, hence, they may be unwilling to commit to sexual relationships which might expose them to be sexually coerced. This finding suggests that the authorities at the JHS level should develop more support structures for YPWDs in schools.

The study also found that those in the coastal zone had higher odds of being sexually coerced compared with their peers in the northern ecological zone. Discussing this within the ecological model [29, 30], a possible reason for this finding could be the normalization of sexual behaviours at the coastal zones [40]. It may be linked with increased risky sexual behaviours among coastal dwellers, increasing the likelihood of YPWDs in the coastal areas to be sexually coerced. However, further research is needed to better understand the risks unique to coastal regions. On the other hand, those belonging to the Islamic religion were less likely to be sexually coerced compared with those with no religion. This finding is perhaps because the Islamic religion frowns upon irresponsible sexual behaviours which deters perpetrators to sexually coerce muslim YPWDs [41, 42].

Akin to the findings of a previous study Mailhot Amborski [15], the study found that the visually impaired had lower odds of being sexually coerced compared to those with hearing impairment. However, this finding contradicts the observation of a previous study [43] which reported a higher likelihood of sexual coercion among individuals living with visual impairment. Previous studies have also found high prevalence of sexual violence against the hearing impaired [15, 36, 44]. The possible explanation is that compared to the visually impaired, the hearing impaired might be unable to easily report or prevent sexual coercive behaviours through communication. In some societies, there is also an erroneous impression that silence in response to certain sexual advances means consent. However, further studies are warranted to advance knowledge regarding these inconsistencies in the literature.

## Strengths and limitations

There are strength and limitations inherent in the study worth acknowledging. First, to the best of our knowledge this is the first study in Ghana to assess the prevalence and factors associated with sexual coercion among YPWDs. Second, the sample size was relatively large. It also included young people from all the major special schools in Ghana. Third, the study was guided by the ecological systems theory [29, 30]. Despite these, the study adopted cross-sectional design, hence the findings can only be interpreted in terms of associations. The same questions were adopted for the various age groups, however, to ensure appropriateness, the wording of the questionnaire items was adjusted for different age categories to make the questionnaire age-appropriate, avoiding confusion among students and ensuring accurate responses, thereby ensuring the validity of the data and subsequent conclusions [34]. Again, sexual coercion was measured with a single item. There are common sexual coercion tactics, including guilt-tripping, threats, for example, breaking up or going elsewhere for sex, emotional blackmail, and giving drugs or alcohol, mainly to lower inhibitions. The study was also limited to only two types of disabilities, hearing impairment and visual impairment. In addition, due to the sensitive nature of the study, there is the possibility of social desirability biases which could lead to either over or underreporting of sexual coercion. In addition, there is the possibility of mistakes in the transcription of the braille version of the questionnaires that were used for the data collection and the general challenges inherent in data collection among persons with disabilities [33, 34, 45, 46].

## Conclusion

The study concludes that there is relatively high prevalence of sexual coercion among in-school YPWDs in Ghana. This is significantly associated with level of education, ecological zone, religion, and the type of disability. The results call for a concerted effort by policy makers such as the Ghana Education Service, Ghana Federation of the Disabled, Ministry of Education, Ministry of Gender, Children and Social Protection to intensify sex education and put in pragmatic steps to halt this problem. A qualitative study is required to gain deeper understanding of sexual coercion among these young people.

## Data Availability

Data will be made available by the corresponding author upon reasonable request.

## Abbreviations

aOR: adjusted odds Ratio
cOR: Crude odds ratio
WHO: World Health Organization
YPWDs: Young people with disabilities
